# The relationship between accuracy in numerosity estimation, math achievement, and math interest in primary school students

**DOI:** 10.3389/fpsyg.2023.1146458

**Published:** 2023-07-10

**Authors:** Leonie Brumm, Elisabeth Rathgeb-Schnierer

**Affiliations:** Department of Mathematics and Natural Science, University of Kassel, Kassel, Germany

**Keywords:** estimation, numerosity estimation, math achievement, math interest, primary education, mathematics

## Abstract

Estimation is a primary activity in everyday life, so getting it “right” in primary school provides a foundational basis in mathematical reasoning. This study focuses on numerosity estimation in primary mathematics, which is one of four types of estimation reported in literature. In numerosity estimation, a non-numerical quantitative representation is typically translated into a number. While it is assumed that fostering numerosity estimation has a great impact on the development of mathematical skills, research indicates that math achievement is influenced by non-cognitive aspects such as students’ math interests. So, math interest could also influence the accuracy in numerosity estimation. In this study, we investigate the relationship between accuracy in numerosity estimation, math achievement, and math interest in third-grade students. For capturing accuracy in numerosity estimation in a standardized way, we developed an online numerosity estimation test. For assessing the construct of math interest, we used an existing questionnaire. Math achievement was assessed by a standardized math test that includes two subtests focusing on arithmetic and application tasks. The sample was comprised of 185 third-grade students. We analyzed the data using correlation and multiple linear regression analysis. The results showed a significant positive correlation between math interest and math achievement. However, no relationship was found between accuracy in numerosity estimation and math interest nor between accuracy in numerosity estimation and math achievement. These partly unexpected findings suggest further studies dedicated to numerosity estimation and its relationship to other constructs.

## 1. Introduction

Estimation is a field of interest for educational and cognitive psychology researchers (Dowker, 2003; Verschaffel et al., 2007). On the one hand, it is an essential part of mathematical cognition, and on the other hand, it bears a solid connection to mathematical procedures (Siegler and Booth, 2005). In addition, estimation is relevant for everyday activities in the lives of children and adults and is an essential core activity in everybody’s life (Siegler and Booth, 2005; Andrews et al., 2021). Recent literature distinguishes four types of estimation: measurement estimation, computational estimation, numerosity estimation, and number line estimation (Sayers et al., 2020). The present study focuses on numerosity estimation and aims to analyze the connection between numerosity estimation and math achievement as well as the relationship between math interest as a non-cognitive process and accuracy in numerosity estimation.

### 1.1. Numerosity estimation

Numerosity estimation is considered one of four types of estimation (Sayers et al., 2020), each of which shares fundamental characteristics. For this reason, we will first define estimation in general and subsequently focus on characteristics that are particularly relevant for numerosity estimation. Overall, estimation can be defined as mental comparison and measurement (Sowder and Wheeler, 1989; Schipper, 2009; Ruwisch, 2014) characterized by “a process of translating between alternative quantitative representations, at least one of which is inexact” (Siegler and Booth, 2005, p. 204). Within all situations and tasks that call for producing an estimate, no exact answer is required, and an approximate answer is sufficient (Sowder and Wheeler, 1989). Estimation may be the most efficient way to solve a given problem if a precise value requires too much time or means (Booth and Siegler, 2006; Albarracín and Gorgorió, 2019).

Numerosity estimation, also known as quantity estimation, refers to estimating discrete quantities (Crites, 1992; Andrews et al., 2021). It “requires translating a nonnumerical quantitative representation (e.g., a visual representation of the approximate volume and density of candies in a jar) into a number” (Siegler and Booth, 2005, p. 198) without resorting to complete counting.

Previous research has shown that while children basically do not estimate adequately, performance improves with age (e.g., Siegler and Booth, 2005; Luwel and Verschaffel, 2008). For example, 6th graders estimate significantly better than 2nd and 4th graders, but no difference occurs between second and fourth graders (Luwel and Verschaffel, 2008). Research has also reported various strategies for estimating a quantity, such as simple arithmetic operations like addition or multiplication while de- and recomposing the quantity to estimate (Siegel et al., 1982; Crites, 1992; Luwel and Verschaffel, 2008). In this sense, a quantity structure needs to be recognized and utilized to estimate, for example by decomposing the quantity to estimate a subset that is followed by recomposing the quantity by using arithmetic operations. Thus, the skill to execute simple arithmetic operations can be essential to performing specific strategies in numerosity estimation. To date, there has been relatively little effort to link numerosity estimation with other constructs such as math achievement and math interest.

### 1.2. Math achievement and estimation

In general, estimation is one essential aspect of learning and understanding mathematics. Sriraman and Knott (2009) consider it a fundamental mathematical skill since estimation activities support the benefit and “development of mathematical concepts (and procedures) and cultivate critical thinking” (p. 210). Similarly, Siegler and Booth (2005) emphasize that estimation requires “going beyond rote application of procedures and applying mathematical knowledge in flexible ways. This type of adaptive problem-solving is a fundamental goal of contemporary mathematics instruction” (p. 197). Accordingly, primary school mathematics should aim to develop students who are flexible problem solvers possessing independent thinking strategies (Siegler and Booth, 2005; Schütte, 2008).

Research literature suggests that estimation accuracy is related to many aspects of mathematical skills (Siegler and Booth, 2005). For example, students who are gifted estimators show better arithmetic skills (Booth and Siegler, 2006) in counting (Barth et al., 2009; Bartelet et al., 2014), number sense (Crites, 1992; Sowder, 1992), and strategy flexibility (Siegler and Booth, 2005; Luwel and Verschaffel, 2008). Furthermore, fostering estimation abilities can greatly impact the development of these skills (e.g., Luwel et al., 2005; Siegler and Booth, 2005). Similarly, Dowker (2003) argues that the effect of estimation can be beneficial to arithmetic achievement because the process of estimation stimulates the development of awareness of number relations as well as resourcefulness with them.

Estimation is a fundamental part of learning mathematics as it plays a decisive role in the acquiring basic arithmetic skills (e.g., Siegler and Booth, 2005; Sayers et al., 2016). Moreover, there is consensus that while estimation is connected to number sense (Verschaffel et al., 2007), the latter is a construct that is difficult to define (Griffin, 2004). One attempt put forward by Sayers and Andrews (2015) conceives foundational number sense as a bundle of number-related essential competencies that require instruction to develop. In this context, estimation is one of eight identified components of foundational number sense (Sayers et al., 2016). Concurrently, foundational number sense is assumed to be fundamental for estimation processes (Siegler and Booth, 2004), as well as for understanding mathematics. The relation between number sense and math achievement is emphasized in literature and empirically supported (e.g., Aubrey and Godfrey, 2003; Aunio and Niemivirta, 2010). For instance, several research projects confirm simple arithmetic competence, another component of foundational number sense (Sayers et al., 2016), to be a strong predictor of later mathematical success (e.g., Malofeeva et al., 2004; Berch, 2005; Jordan et al., 2007; Ivrendi, 2011). As mentioned above, number sense can also be fundamental in performing numerosity estimation. Generally, it is widely believed that estimation is a determinant of later arithmetical achievement (Siegler and Booth, 2004; Sayers et al., 2016; Andrews et al., 2021).

The association between estimation and math achievement has been addressed in several studies (e.g., Siegler and Booth, 2004; Booth and Siegler, 2006; Schneider et al., 2009; Sasanguie et al., 2013; Wong et al., 2016) that have predominantly concentrated on number line estimation (Schneider et al., 2009; Sasanguie et al., 2013). Numerosity estimation has also been addressed by various studies that either address its relation to a single aspect of math achievement or examine a specific type of task related to math achievement. Results of such studies suggest that counting is fundamental to a successful process of solving numerical estimation tasks (Lipton and Spelke, 2005; Barth et al., 2009). Barth et al. (2009) report that the accuracy of children’s estimates depends on their counting ability (Barth et al., 2009). Kindergarten students’ efficiency in numerosity estimation explains, besides counting and comparing, a unique part of the variance in arithmetic achievement in first grade but no significant association between estimation and arithmetic achievement was found (Bartelet et al., 2014). Besides, according to their research, Wong et al. (2016) suggest that sole contributions of diverse estimation abilities affect arithmetic achievement for six-year-old children. Measures of numerosity estimation predict arithmetic achievement (Wong et al., 2016). Booth and Siegler (2006) report a positive correlation between judging numerosities and math achievement in second and third graders.

A substantial body of research focuses on the relation between numerosity estimation and single aspects of math achievement or between one specific type of task in numerosity estimation and math achievement. Although the literature often outlines a connection between numerosity estimation and math achievement, there is only limited empirical evidence of whether math achievement and accuracy in numerosity estimation regarding different types of perception tasks are related. Consequently, the relation between numerosity estimation and its impact on math as well as arithmetic achievement is not well understood.

### 1.3. Math interest and estimation

In recent decades, educational and psychological research has increasingly studied the influence of interest on learning and development in various educational settings (Krapp, 2002). In this vein, most researchers distinguish between individual/personal interest and situational interest (e.g., Krapp, 2002; Hidi and Renninger, 2006). Individual interest is a relation between a student and a specific content (e.g., Krapp, 2002; Hidi and Renninger, 2006) that is relatively stable and well-developed (e.g., Krapp, 2000; Hidi and Renninger, 2006; Jansen et al., 2016). Situational interest can be understood as a short-term affective engagement caused by external factors (e.g., Hidi, 1990; Krapp, 2002; Hidi and Renninger, 2006). Furthermore, interest is an affective, motivational construct linked to intrinsic motivation (e.g., Köller et al., 2001; Krapp, 2002, 2007; Renninger et al., 2002; Aunola et al., 2006).

Research findings and reviews suggest that interest contributes significantly to performance (e.g., Hidi and Harackiewicz, 2000; Krapp, 2002; Lee et al., 2014; Trautwein et al., 2015). For example, higher interest supports engagement and persistence in completing school tasks (e.g., Eccles and Wigfield, 2002; Renninger et al., 2002). In addition, an activity based on interest can include enjoyment and involvement, which can enhance attention, concentration, and positive affect, which in turn influence any learning process (Eccles and Wigfield, 2002; Hidi, 2006). This reported research also implicates an effect of math interest on numerosity estimation accuracy. It may be assumed that for instance higher attention and concentration during an estimation can also have an influence on estimation accuracy by capturing and observing the quantity to be estimated in a concentrated way. For example, structures within the quantity could be identified that can be used for the estimation process. Consequently, the estimation process influences the estimation accuracy. However, previous studies have not investigated the influence of non-cognitive processes like math interest on accuracy in estimation in general or numerosity estimation in particular. Although a broad body of studies concludes that non-cognitive processes have a differential impact on math achievement (e.g., Hattie, 2009; Lee and Shute, 2010; Jansen et al., 2016), no clear link has been established between numerosity estimation and math interest.

While the early school years are essential for learning mathematics and developing math interest, only a few studies of estimation have focused on preschool and primary school students (Aunola et al., 2006). In such studies, findings on the connection between math interest and math achievement are inconsistent. For example, Fisher et al. (2012) reported a positive relationship between math achievement and math interest in preschool students, and subsequently students’ initial achievement levels predicted their later level of interest in math tasks. In the same way, early math interest predicted later math achievement. They suggest that this “reciprocal relationship […] has already begun by preschool” (Fisher et al., 2012, p. 679). In contrast, Gottfried (1990) found a significant relation between young children’s intrinsic motivation and their math achievement, but their motivation did not predict standardized achievement test scores. One study of students from kindergarten through 12th grade reported that among non-cognitive constructs, motivation and interest are related to students’ academic achievement (Lee and Shute, 2010). In contrast, however, Lee and Chen (2019) report that their cross-country study found in only 20% of the tested countries, interest had “a moderately strong predictive power for math achievement (r ≥ 0.224) […] at the fourth grade” (p. 8). Across all countries, the average correlation between interest and math achievement was weak in the fourth grade (Lee and Chen, 2019), but it was consistently stronger in secondary school (Lee and Chen, 2019). Indeed, for that matter, the overall results from Aunola et al. (2006) and Viljaranta et al. (2009) showed a cumulative developmental cycle between children’s mathematical and arithmetic performance and math-related task motivation in primary school (Aunola et al., 2006). A cumulative developmental cycle means the higher the performance, the more math-related task motivation later, which predicted further math performance (Aunola et al., 2006; Viljaranta et al., 2009). They “use the term task motivation to refer to children’s interest value or intrinsic motivation” (Aunola et al., 2006, p. 23).

In summary, research focusing on the relationship between math interest and math achievement is ambivalent, but multiple findings suggest that interest contributes significantly to various aspects of a learning process that lead to better performance. If the assumption that numerosity estimation and math achievement are related is correct, then the relationship between achievement and interest raises the question of whether there is also a relationship between math interest and accuracy in numerosity estimation on the condition that students consider estimation as part of mathematics. Even though estimation is considered an essential aspect of learning and understanding mathematics, there is currently no research literature about the relationship between math interest or other non-cognitive processes and accuracy in numerosity estimation.

### 1.4. Aims and research questions

The current study examined the relations between accuracy in numerosity estimation, math achievement, and math interest. Particularly, this project investigated the relationship between math achievement and accuracy in numerosity estimation as well as the influence of accuracy in numerosity estimation on math achievement. In addition, the relationship between math interest and accuracy in numerosity estimation was analyzed. Accordingly, two main research questions and associated hypotheses were examined:

1. To what extent is there a relationship between math achievement and estimation accuracy for two- and three-dimensional numerosity estimation tasks in third grade?

In the literature, numerosity estimation and math achievement are assumed to be related. Previous research has shown that numerosity estimation influences arithmetic achievement (Wong et al., 2016). Booth and Siegler (2006) found that there is a relationship between one specific type of numerosity estimation task and math achievement in second and third grade. Therefore we expect:

*H_1_*: Math achievement and estimation accuracy for two- and three-dimensional numerosity estimation tasks are positively related.

Concerning our second focus:

2. To what extent is there a relationship between math interest in third-grade students and their accuracy in numerosity estimation tasks?

As already addressed in the first research question, a relation between numerosity estimation accuracy and math achievement is assumed and has been tentatively confirmed in terms of specific tasks or mathematical partial aspects (Booth and Siegler, 2006; Wong et al., 2016). A reciprocal relationship between children’s math achievement and math interest is already supposed to start in preschool and a positive link has already been established (e.g., Fisher et al., 2012; Jansen et al., 2016). Since numerosity estimation is a mathematical activity, and provided that (1) math interest and math achievement as well as (2) math achievement and accuracy in numerosity estimation are positively related, we expect:

*H_2_*: Math interest and numerosity estimation accuracy for two- and three-dimensional numerosity estimation tasks are positively related.

## 2. Materials and methods

### 2.1. Participants and procedure

This project sampled 185 third-grade student volunteers who returned a parent-signed consent form. Additionally, we obtained teachers’ and head teachers’ consent. Student ages ranged from 8.7 years to 11.1 years (*M* = 9.5, *SD* = 0.4). Ninety-four students were girls (approximately 51%), and 91 were boys (approximately 49%). Students came from 13 classrooms in five German public schools, composed of two city schools, one suburban school, and two village schools. About 88% of the students were born in Germany, and about 74% used German as their first language.

Since we wanted to avoid counting and did not focus on quasi-simultaneous acquisition, we chose a number range larger than 30. In the second grade in Germany, the number range is extended from 20 to 100. By choosing the third grade, we could be sure that the number range had already been extended to 100 and that a student’s lack of number range development could not be the main reason for an inappropriate estimate. Tasks in the number range 100–150 were included to create challenging tasks and to be able to better differentiate estimation accuracy.

To answer the research questions, we designed a quantitative study and collected data using a digital standardized test to determine estimation accuracy, a digital questionnaire to measure non-cognitive interest in mathematics, and a standardized test of math achievement. The data collection took place during school hours from May 2022 to July 2022. The digital numerosity estimation test and digital questionnaires were administered in one lesson (45 min) per class on the same day. We conducted the standardized paper-pencil-test (45 min) to measure math achievement on another day.

### 2.2. Measures

#### 2.2.1. Numerosity estimation accuracy

To our knowledge, no published instrument reliably measures accuracy in numerosity estimation in a standardized way. For this reason, we developed an online numerosity estimation test for primary school students.

Numerosity estimation means estimating the number of structured or unstructured elements in a bounded collection without exact counting. Therefore, 21 estimation tasks were “presented in such a way as to preclude exact counting of the items” (Hogan and Brenzinski, 2003, p. 260). To avoid counting as an entire strategy, each task (i.e., picture of a number of elements) was presented for 20 s (Luwel and Verschaffel, 2008). The quantities in the number range of 31–144 also hindered counting all elements (Albarracín and Gorgorió, 2019). Such a range of quantities is realistic to estimate for third-grade students because the number ranges have already been introduced. In addition, this test did not include tasks requiring knowledge external to estimation, so this would not affect estimation accuracy. After the picture disappeared, students had 40 s more to adjust their results with a slider. Independently from the items, they could adjust their estimates between zero and 500 on the slider scale. We chose this uniform response format for all items so that the estimates would not be affected by different number ranges of the slider scale and to counteract errors that could occur when using a keyboard to type in the result (e.g., numbers are not typed in according to place value). To adjust the slider, only the computer mouse was needed. The handling was comparatively intuitive. Under the slider, the selected number was visible during the adjustment of the slider and finally, the result could be read. Altogether, students had 1 min to estimate each of 21 items.

**Figure 1:**
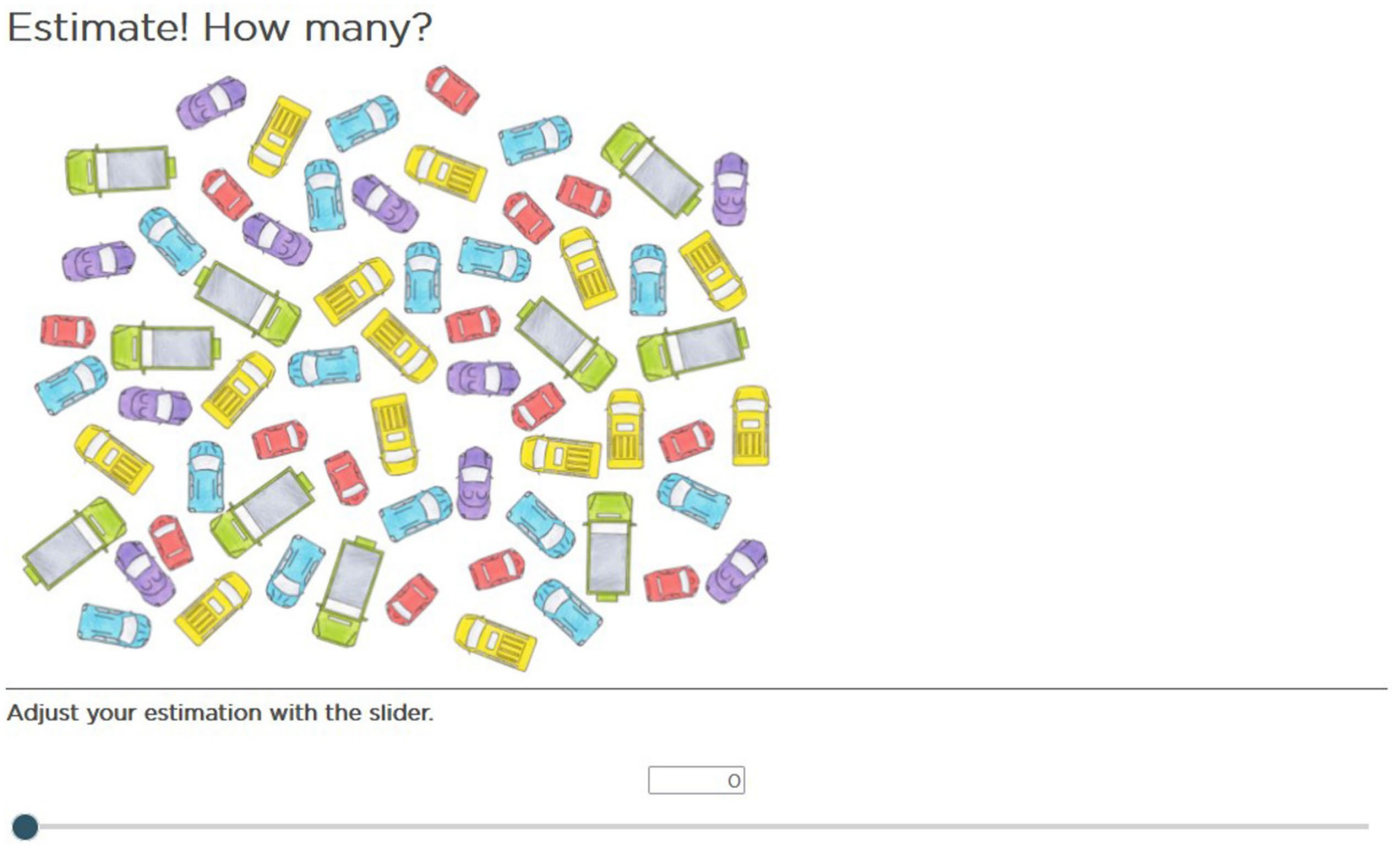
Example item: two-dimensional, unstructured, unequal items.

To ensure content validity, the test items were constructed to represent a broad range of numerosity estimation tasks. Items featured a range of task characteristics: dimension (two- or three-dimensional), the arrangement of the elements (structured or unstructured), and the nature of the elements (equal or unequal regarding size, shape, and color). Some items were created based on the items Luwel and Verschaffel (2008) used in their study. The images of two-dimensional numbers have shown, for example, iconic cars, coins or animals. Figure 1 shows an example item of a two-dimensional quantity with unstructured arrangement and unequal items (the cars have different sizes, shapes, and colors).

**Figure 2:**
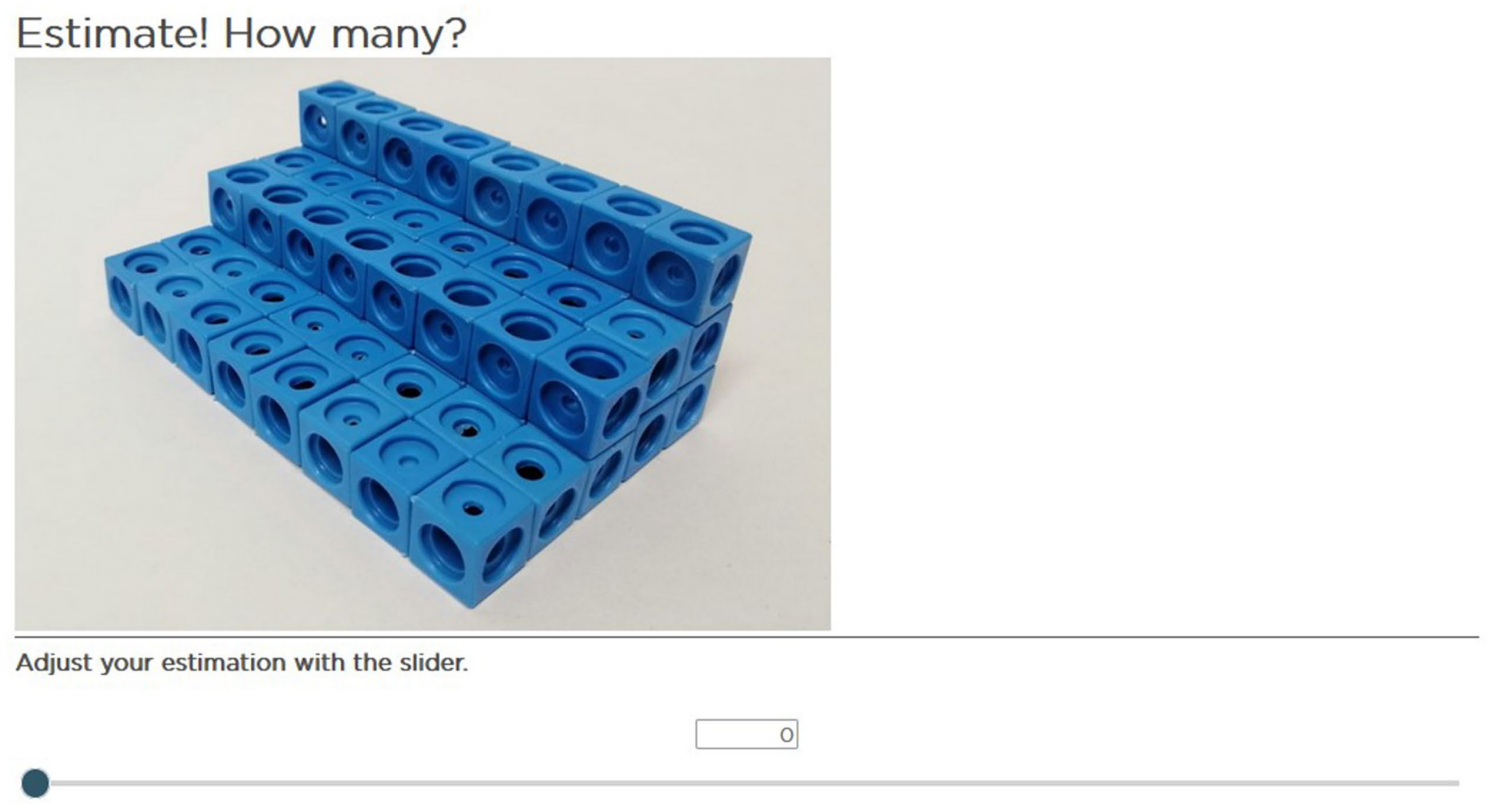
Example item: three-dimensional, structured, equal elements.

Finally, it is essential to regard that on a screen, it was only possible to see a representation of a three-dimensional quantity, which means that students must perceive the number of elements as three-dimensional in the first step of the estimation. That is, they must realize that some elements were behind those in front and could not be seen. The pictures of the three-dimensional numbers have shown, for example, real building bricks, plug cubes, or wooden cubes. Figure 2 displays an example item of a three-dimensional quantity with structured arrangement and equal elements.

Before the test started, we introduced the term “estimation” and gave instructions on the test procedure and the response format of the items. In a pretest, students worked on two test items and had the opportunity to ask questions about the test procedure. After this introduction phase, the actual test started.

The value for any item in the numerosity estimation test reflected a standardized absolute deviation, calculated as follows:


$$\frac{\text{Actual value}-\text{Estimated value}}{\text{Standard deviation}\;\left(\begin{array}{l} \text{in relation to acutal value}\\ \text{instead of mean value} \end{array}\right)}.$$

We used this standardization because as the number of elements in an item increases, so too does the standard deviation of estimates. Since the number of elements to be estimated influences the mean value, we calculated the standard deviation concerning the actual value. As relative errors occur more frequently with small numbers than large ones, the raw values or the absolute deviation would not be comparable due to the different number ranges of the numbers to be estimated. Standardizing all values in this way made response estimates comparable for data analysis.

In relation to testing evaluation, no values were excluded except 0 and 500, which correspond to the minimum and maximum values on the slider. Hence, we assumed these values are not valid. Using the study sample, we measured the instrument’s construct validity with exploratory factor analysis (principal axes factor analysis; Brandt, 2020; Moosbrugger and Kelava, 2020). Four factors resulted. We eliminated three of the 21 items due to similar double loading or content fit. Thus, at least four items form a construct and the items were not strongly reduced with respect to content validity (Brandt, 2020). All items in one factor were tested again within a principal component analysis to ensure only one factor was within. Consequently, the reliability of one scale of items of one factor was measured by Cronbach’s Alpha. In addition, we examined the corrected item-total-correlation in each subscale of the numerosity estimation test. Item-total-correlations between 0.4 and 0.7 are considered good (Kelava and Moosbrugger, 2020). Within the scales *SmallN*, *Mix* and *3DlargeN* the corrected item-total-correlation of each item was sufficiently large (≥0.4). For the scale *2DlargeN*, the corrected item-total-correlation of three items was between 0.47 and 0.55. One item had a corrected item-total-correlation of 0.37. Since this value was close to a good value, the item is theoretically significant for the characteristic of interest, and the reliability does not change significantly by removing this item, the item was not removed. In summary, the exploratory factor analysis for construct validation revealed four dimensions and in line with the item-total-correlations one-dimensionality within these four dimensions (Moosbrugger and Kelava, 2020). Good item-total-correlations provide indications that the items measure the same characteristic in terms of content (Kelava and Moosbrugger, 2020).

Subsequently, we computed factor scores as means over all values of items measuring one similar factor because the factor loadings per factor did not differ too much. The means of the four scales represent a standardized mean of deviation from the numerosity to be estimated: the smaller the deviation, the higher the estimation accuracy. Table 1 shows the resulting four scales incorporating the factor scores as measures for accuracy in numerosity estimation.

**Table 1:** Estimation scales.

Scale	Items	Number range <50/50–100/>100	Dimension 2D/3D	Arrangement structured/unstructured	Cronbach’s alpha (α)
SmallN	6	6/0/0	4/2	3/3	0.78
2DlargeN	4	0/1/3	4/0	3/1	0.69
Mix	4	1/2/1	2/2	1/3	0.75
3DlargeN	4	0/1/3	0/4	2/2	0.71

The first scale *SmallN*, with six items, represents items in the number range from 31 to 45 and had a reliability of α = 0.78. Scale *2DlargeN* shows a reliability of α = 0.69 and includes four items in the number range from 82 to 132. All items were two-dimensional in this scale. *2DlargeN* was the only scale that has questionable reliability. However, the value is very close to a satisfactory value, and the items in this scale were meaningful in terms of content, which is why this scale was included in further analyses. The third scale, *Mix*, also comprised four items with a reliability of α = 0.75. Two items in *Mix* were in the numerical range of around 70, one contained 123 elements to estimate, and another showed 42 elements that were arranged three-dimensionally and unstructured. In this scale, two items were arranged in an unstructured way, and two were arranged in a structured way. Finally, scale *3DlargeN*, with four items and reliability of α = 0.71, represents items belonging to the number range from 72 to 144. All were three-dimensional items. In summary, the estimation accuracy measure was comprised of four scales, each with alpha 0.69–0.78, and consisting of four to six items.

#### 2.2.2. Math achievement

For assessing math achievement, we administered a timed, standardized test for third graders (May and Bennöhr, 2021). The test was presented in a booklet and consisted of 74 items organized into two subtests. The first subtest, with 56 items, focused on basic knowledge of arithmetic and computational abilities. For this reason, we refer to this subtest as arithmetic achievement. The following contents were in the arithmetic subtest:

- Subtraction and addition [e.g., the following tasks are to be solved: “48 − 34 + 35” (May and Bennöhr, 2021, p. 4) or “Which number is 450 greater than 240?” (May and Bennöhr, 2021, p. 12)].
- Understanding of place value in combination with subtraction and addition [e.g., “You have four hundreds and take away 12 tens. How many whole hundreds do you have left?” (May and Bennöhr, 2021, p. 6)].
- Completing number series [e.g., this series of “50, 46, 42, 38” is to be supplemented by two further numbers (May and Bennöhr, 2021, p. 8)].
- Acquisition of the number of small cubes in a three-dimensional figure (that exists of small cubes).
- Solving an arithmetic square and arithmetic triangle.
- Inserting appropriate arithmetic symbols.
- Numberline estimation.

The second subtest comprises 18 application-oriented tasks (application task achievement), such as:

- Solving word problems [e.g., students solve: “24 children come to the sport. Six children are in one group. How many groups are there?” (May and Bennöhr, 2021, p. 18)].
- Extracting information from a table.
- Reading a temperature scale.
- Number registration of small cubes needed to make at the end a big cube with the structure of 3 × 3 × 3 small cubes (12 small cubes are already rudimentarily represented in the structure of the bigger cube).
- Reading the weight displayed on a scale.

Including the introduction and instruction, the test lasted about 45 min, corresponding to a standard lesson in German classrooms. The test was conducted in a classroom with all students in one class.

The test was evaluated in such a way that one point was given for each correctly answered item. It was therefore possible to achieve a total of 74 points if all items of the test were answered correctly. Consequently, a sum score was calculated for each student from the correctly answered items. The reliability of the whole test was α = 0.93 (May and Bennöhr, 2018), with α = 0.91 for the first subtest (arithmetic achievement) and α = 0.81 for the second subtest (application task achievement). Therefore, the reliability of the test can be considered excellent (Döring and Bortz, 2016).

#### 2.2.3. Math interest

For measuring math interest, we used two published questionnaires from large-scale comparison studies. First, the scale “Interesse an Mathematik” (interest in mathematics) was derived from the IGLU 2001 questionnaire survey (Bos et al., 2005), and the second came from the national questionnaire of the PISA 2012 “INTMAT—Freude und Interesse an Mathematik” (interest in and enjoyment of mathematics; Mang et al., 2018). The scale “interest in mathematics” with five items, slated initially for students at the end of fourth grade, showed a reliability of α = 0.75 (Bos et al., 2005). In our sample, the reliability of this instrument was α = 0.76. The five items test math interest in different areas. For example, the children must answer the item “I find it exciting to discover rules or tricks in mathematics myself.” The other scale “interest in and enjoyment of mathematics” (INTMAT) with four items showed a reliability of α = 0.89 for 15-year-old students (Mang et al., 2018). These items were linguistically appropriate for third-grade students and we obtained a reliability of α = 0.85 with our sample. “I look forward to my math lessons” is, for example, one of the four items.

We adapted the response options from the PISA 2012 study to the options of IGLU 2001 and used a four-point Likert format for students’ responses within both scales. The response options were “not correct” (coded as 1), “partly correct” (coded as 2), “almost correct” (coded as 3), and “correct” (coded as 4). Generally, we read every sentence out loud to the class before students answered the item.

Since both scales showed an almost identical mean value and median (Interest in mathematics: *M* = 3.07, *Mdn* = 3.20; INTMAT: *M* = 3.04, *Mdn* = 3.25) as well as an identical standard deviation (*SD* = 0.79), we decided to merge the two scales together for further analyses. The reliability of all items of both scales is α = 0.88 (9 items). Further analyses were calculated using the mean of the responses of the nine items and refer to this derived scale as “math interest.”

### 2.3. Statistical analysis

For statistical analysis, we used the sum scores of the obtained points in the math achievement test, the mean of the two interest scales, and the mean of each of the four scales of the numerosity estimation test.

The analyses were calculated using SPSS Statistics. To examine the relationship between accuracy in numerosity estimation, math achievement, and math interest in third grade, descriptive and correlational analyses were used. In addition, we performed a multiple linear regression analysis to examine the predictive capacity of math interest, the four scales of accuracy in numerosity estimation, and gender (male coded 0, female coded 1) on math achievement as the criterion variable. Since research has repeatedly shown that gender can also have an impact on mathematical competencies (Else-Quest et al., 2010; Keller et al., 2022), gender was included as independent variable. In German primary schools, boys often show slightly higher competencies than girls (Stanat et al., 2022).

## 3. Results

### 3.1. Descriptive results

Descriptive statistics for each of the four numerosity estimation scales are presented in Table 2, which shows the mean and standard deviation for each scale.

**Table 2:** Descriptive statistics of estimation scales.

	*M*	*SD*
SmallN	0.59	0.56
2DlargeN	0.87	0.37
Mix	0.71	0.53
3DlargeN	0.85	0.40
SmallN_Pd	36.83%	41.47%
2DlargeN_Pd	40.13%	17.05%
Mix_Pd	38.67%	30.29%
3DlargeN_Pd	41.88%	19.40%

Pd, Percentage deviation from the actual value.

Recall that the mean for estimation accuracy derived from the standardized absolute deviation from the actual value (see section 2.2.1). Because of the standardization, these values are comparable and represent numerical/value proximity; the smaller the mean, the better the estimation accuracy. Thus, in scales *2DlargeN* and *3DlargeN*, students’ estimates deviated most from the actual value in comparison to the other two scales. On the one hand, *2DlargeN* contains items that represent two-dimensional quantities in the number range of 82–132, and on the other hand, *3DlargeN* includes items that represent three-dimensional quantities in a slightly larger number range of 72–144. The mean was almost identical for these two scales. According to Table 2, the children’s estimates deviated least from the actual values in *SmallN*, which reflects the number range 31–45. Since the standardized values of estimation accuracy are difficult to interpret, the mean and standard deviation of the percentage deviation from the actual value are also displayed in Table 2.

Table 3 displays students’ mean sum scores in the math achievement test, the means of both subscales, and the mean for math interest. The mean sum score corresponds to the average number of correct items. For the sample, about 63% of the tasks of the math achievement test were solved correctly. In the arithmetic achievement subtest, students solved an average of 67% of the tasks correctly. In comparison, application tasks including word problems proved more difficult, with only 52% of these tasks solved correctly. Table 3 also presents the mean and standard deviation for math interest. The maximum value for math interest would be 4, and 3.05 is the mean value in our sample.

**Table 3:** Descriptive statistics of math achievement and math interest.

	Items	*M*	*SD*
Math achievement	74	46.75	11.34
Arithmetic achievement	56	37.62	8.62
Application task achievement	18	9.32	3.62
Math interest	9	3.05	0.69

### 3.2. Relationship between numerosity estimation accuracy and math achievement

Table 4 displays results from our correlation analysis. Although there were a few negative weak correlations, they were not statistically significant. A negative relationship means that the higher the math achievement, the lower the standardized absolute deviation (i.e., close proximity). Regarding math achievement in total, there was only a weak negative correlation with scale *SmallN* (*r* = −0.13, *p* > 0.05). Furthermore, this scale also correlated weakly and negatively with both subscales of the math test. Focusing on the subscales of the math achievement test, there was also a weak correlation between scale *Mix* and achievement in application tasks (*r* = −0.11, *p* > 0.05), but no correlation with arithmetic achievement or math achievement in general. It is noticeable that all Pearson coefficients were negative except between scale *2DlargeN* and arithmetic achievement (*r* = 0.02, *p* > 0.05) as well as *3DlargeN* and arithmetic achievement (*r* = 0.03, *p* > 0.05).

**Table 4:** Correlations between numerosity estimation accuracy, math achievement and math interest.

	1	2	3	4	5	6	7	8
1. SmallN	--							
2. 2DlargeN	0.08	--						
3. Mix	0.49^**^	0.10	--					
4. 3DlargeN	0.33^**^	0.41^**^	0.27^**^	--				
5. Math achievement	−0.13	−0.01	−0.09	−0.00	--			
6. Arithmetic achievement	−0.11	0.02	−0.09	0.03	0.94^**^	--		
7. Application task achievement	−0.14	−0.03	−0.11	−0.08	0.78^**^	0.64^**^	--	
8. Math interest	−0.05	−0.04	−0.17^*^	0.03	0.21^**^	0.19^**^	0.21^**^	--

^*^p < 0.05. ^**^p < 0.01.

We conducted a multiple linear regression analysis, with math achievement as the dependent variable, to investigate how the residuals of estimation accuracy, math interest and gender predict math achievement. To test the model’s assumptions, high multicollinearity had to be ruled out (Urban and Mayerl, 2011). For this reason, the correlations between the independent variables and the variance inflation factor were calculated. The results show no high correlation between the scales of estimation accuracy and no high correlation between these scales and math interest (Table 4). In addition to the results of the correlation analysis, the resulting variance inflation factors between 1.01 and 1.40 confirm that there is no multicollinearity for the independent variables mathematics, gender, *SmallN*, *2DlargeN*, *Mix*, *and 3DlargeN*. Accordingly, this requirement for regression analysis was fulfilled (Urban and Mayerl, 2011). We also tested whether the residuals are normally distributed and checked for homoscedasticity using the Breusch-Pagan test. The normal distribution of the residuals and homoscedasticity were confirmed. Table 5 shows the results of the multiple linear regression analysis.

**Table 5:** Regression model: contribution to math achievement.

Variable	Unstandardized *b*	Standardized *β*	Standard error	*t*
Constant	39.82^***^		4.62	8.62
Math interest	3.20^**^	0.20^**^	1.20	2.67
Gender	−3.85^*^	−0.17^*^	1.63	−2.37
SmallN	−2.80	−0.14	1.72	−1.63
2DlargeN	−0.231	−0.01	2.40	−0.10
Mix	−0.12	−0.01	1.79	−0.07
3DlargeN	1.22	0.04	2.39	−1.63
*R^2^*	0.09			
Adjusted *R^2^*	0.06			
*F*(*df* = 6, 178)	2.87^*^			

^*^p < 0.05; ^**^p < 0.01; ^***^p < 0.001.

The results showed that the overall regression model explains 6% of total variances in math achievement, *F*(6, 178) = 2.87, *p* < 0.05, and revealed that accuracy in numerosity estimation does not significantly predict math achievement.

### 3.3. Relationship between numerosity estimation accuracy and math interest

To test our second hypothesis, we firstly analyzed the relationship between math achievement and math interest of third-grade students using Pearson’s *r*. We examined math interest and math achievement with both subscales, arithmetic achievement, and application task achievement (see Table 4).

The results show a significant positive correlation between math achievement and math interest (*r* = 0.21, *p* < 0.01). There was a similar significant correlation between math interest and both subscales of math achievement. The regression analysis with math achievement as the criterion variable showed that math interest [*β* = 0.20, *t*(178) = 2.67, *p* < 0.01] was a significant predictor of math achievement for third graders.

For testing our second hypothesis (H_2_), we analyzed the relationship between estimation accuracy and math interest. The results of this Pearson correlation analysis are presented in Table 4. There was no correlation between math interest and the scales *SmallN*, *2DlargeN*, and *3DlargeN*. However, there was a weak negative, significant correlation between the estimation scale *Mix* that represents different two- and three-dimensional estimation tasks and math interest (*r* = −0.17, *p* < 0.05). A negative relationship, in this case, means that the higher the math interest, the lower the standardized absolute deviation, thus the better the estimation accuracy.

## 4. Discussion

This paper analyzed the relationship between math achievement, math interest, and accuracy in numerosity estimation within different types of tasks for third graders. We targeted the field of numerosity estimation, which has been shown to be important for the development of math skills (Crites, 1992; Sowder, 1992; Booth and Siegler, 2006; Luwel and Verschaffel, 2008). Our discussion relates our findings to the current research literature on developing estimating competence in students.

### 4.1. Accuracy in numerosity estimation, math achievement, and math interest

We analyzed accuracy in numerosity estimation, operationalized as the standardized deviation from the actual value or quantity to be estimated. As we expected, students’ estimation accuracy was highest in *SmallN* in relation to the other scales of estimation. This scale contained the smallest numbers to be estimated in comparison to the other estimation scales. The standard deviation within this scale was comparatively high. This may be because no estimates were excluded. In comparison, estimates of *2DlargeN* and *3DlargeN* were less accurate. One possible explanation for this pattern could be the increased number range covered by these two scales, because they are very similar and relatively large, representing three quantities in the number range > 100 and one quantity in the number range between 50 and 100. It is interesting to note that accuracy in numerosity estimation was relatively similar for both scales, although the quantities in scale *2DlargeN* were displayed in two-dimensional representations, whereas scale *3DlargeN* included three-dimensional representations. It would be reasonable to assume that third-grade students were more familiar with two-dimensional mappings (e.g., a hundred field) and thus may have developed more benchmarks in two-dimensional mappings. In addition, it could be argued that third graders have already encountered two-dimensional “bundles” of elements, which enabled fast coding or spontaneous structuring of two-dimensional quantities. However, while such structuring could have led to a closer proximity to (lower deviation from) the actual value, and contrary to our expectations, that was not the case.

Many qualitative studies of numerosity estimation have analyzed estimation accuracy as the percentage deviation from the real value (Siegel et al., 1982; Crites, 1992; Luwel and Verschaffel, 2008). To compare our results on accuracy with other studies, we reported the mean values of the percentage deviations. However, we assumed that the number of elements to be estimated has a crucial influence on the estimate and, accordingly, the adequacy of an estimate should not be measured only by the percentage deviation. To correct for this tendency, we used the standardized absolute deviation. In the relevant literature, an adequate estimate has often been assessed using different limits or ranges for the percentage deviation from the actual value. For example, Luwel and Verschaffel (2008) defined a limit of up to 10% as a very good estimation and the range between 10% and 25% deviation as a good estimate. Older studies have defined a limit of up to 50% as an adequate estimate (Siegel et al., 1982; Crites, 1992). Regarding the mean of the percentage deviation from the actual value for the four estimation scales in our study, we reasoned that most students did not estimate adequately, according to the less liberal 25% guideline of Luwel and Verschaffel (2008). At the same time, our data agreed with Luwell and Verschaffel’s conclusion that primary school children have trouble making adequate estimations. This is also in line with Siegler and Booth (2004) who have argued that low estimation skills in second and fourth graders suggests “a lack of number sense and conceptual structures” (p. 429). Altogether, our students’ low estimation accuracy was not an unexpected result, perhaps because estimation is not a very prominent topic in instruction (Luwel and Verschaffel, 2008; Sayers et al., 2020; Andrews et al., 2021). In our opinion, estimation should be considered a learning object, with the ability to adequately estimate addressed through instruction, so that students learn to estimate reflectively, according to estimation task characteristics (Wessolowski, 2014).

Regarding the results of the math achievement test, our third graders performed better on the arithmetic achievement subtest than on the subtest involving application tasks. This is not surprising, because arithmetic is a highly focused subject of instruction (National Council of Teachers of Mathematics, 2000). In the descriptive results, the mean value of math interest was 3.05 on a scale with maximum value of 4.0. The results of the IGLU 2001 study, from which the math interest questionnaire originated, also showed a high mean level of mathematics interest for the end of fourth grade (Walther et al., 2003). In general, primary school students often exhibit relatively high interest (Helmke, 1993), which we can confirm with our data.

### 4.2. Relationship between accuracy in numerosity estimation and math achievement

Siegler and Booth (2005) have described a solid connection between competence in estimation and mathematical procedures. In this vein, researchers often assume that estimation ability is a determinant of later math performance, particularly arithmetic achievement (Siegler and Booth, 2005; Sayers et al., 2016; Andrews et al., 2021). Few quantitative studies exist that have investigated the relationship between math achievement and numerosity estimation (Booth and Siegler, 2006; Bartelet et al., 2014; Wong et al., 2016). In contrast with those studies, we investigated this relationship by testing numerosity estimation accuracy of third-grade students across different types of perception tasks: either two- or three-dimensional, utilizing different number ranges, with structured or unstructured elements.

In relation to the research questions, our results showed no significant relationship between math achievement of third-grade students and their accuracy in numerosity estimation. It is noteworthy that all but two of the correlation coefficients were negative, since a negative correlation means that the lower the deviation from the actual value, the higher the estimation accuracy. From these results, we conclude that estimation skills do not develop automatically, even in students with high math achievement. Accordingly, those who performed very well on the math achievement test did not automatically achieve a low deviation from the actual value. Thus, we cannot confirm the results from Booth and Siegler (2006) who report a positive correlation between judging numerosities and math achievement in third graders. In their study, they invite the students to estimate which number, out of two, corresponds to the number of candies shown in a container. The different results could therefore be due to the different measures for numerosity estimation and math achievement. Finally, the multiple linear regression analysis revealed that none of the four scales measuring accuracy in numerosity estimation significantly predicted math achievement. Based on these results, we cannot confirm the theoretical assumption that estimation accuracy determines math achievement. Such a conclusion is tentative, however, because we examined the relationship with only third-grade students, using a very particular methodology.

In general, our pattern of results is surprising. After all, a relationship between estimation accuracy when estimating smaller quantities (<50) and math achievement was expected. Structuring the elements, decomposing them, and then recomposing them using simple arithmetic competencies would have been one possible strategy for estimating these numbers (Siegel et al., 1982; Crites, 1992; Luwel and Verschaffel, 2008). We would also have expected a connection with two-dimensional presentation of numbers since those representations are certainly used in math lessons to visualize quantities (e.g., a field of hundreds as a square field of points), and structuring would have been conceivable. Because both estimation and simple arithmetic competence are components of the foundational number sense (Sayers et al., 2016), which in turn is linked to math achievement (e.g., Aubrey and Godfrey, 2003; Aunio and Niemivirta, 2010), we had not expected these results and cannot verify our hypothesis (H_1_).

The characteristics of the math achievement test may be one explanation for the fact that the estimation accuracy was not correlated to math achievement in our study. In the math achievement test, flexible approaches and the understanding of the non-symbolic magnitude was hardly addressed. There was only one task in which a non-symbolic magnitude was to capture. But, for solving an estimation task successfully, students “need to have an approximate understanding of the non-symbolic magnitude expressed by the symbolic magnitude” (Bartelet et al., 2014, p. 15). The math achievement tasks focused on precisely applying specific mathematical procedures. In this regard, it is important to consider that estimation is not linked to any specific mathematical procedure but requires flexible approaches and flexible use of already acquired knowledge (Siegler and Booth, 2005; Schütte, 2008). Furthermore, counting is fundamental for numerosity estimation (Barth et al., 2009) and an important mathematical skill (Luwel et al., 2005; Siegler and Booth, 2005; Luwel and Verschaffel, 2008). However, due to the structure of the math achievement test for the third grade, counting skills were not queried. Apart from this, the analyses should be performed again with a larger sample to verify these results. Finally, high ceiling could have influenced student performance on the *2DlargeN* and *3DlargeN* scales. These could have been caused by the items being too difficult for the students, which should be considered before the test is administered again.

### 4.3. Relationship between accuracy in numerosity estimation and math interest

Previous findings suggest that interest as a non-cognitive aspect contributes significantly to various aspects of a learning process that lead to better learning results (e.g., Hidi and Harackiewicz, 2000; Krapp, 2002; Lee et al., 2014; Trautwein et al., 2015). But previous research and resulting conclusions about the connection between math interest and math achievement have been inconsistent. Overall, most studies have focused on students in higher grades. In that context, there exists a possible connection between estimation and general math ability (Siegler and Booth, 2005) that can be influenced by math interest (e.g., Aunola et al., 2006; Fisher et al., 2012). For this reason, we investigated the relationship between math interest and math achievement first and then subsequently the relationship between math interest and accuracy in numerosity estimation.

We found a weak, positive, significant relationship between math achievement and math interest for students in third grade. The results of our correlational analysis accord well with Lee and Chen (2019) who found a weak correlation between math achievement and math interest in fourth grade. It seems to be important to promote the interest of students, because there can be a stronger relationship later between interest and math achievement in secondary school (Lee and Chen, 2019). Furthermore, the multiple linear regression analysis showed that math interest predicts math achievement on a significant level.

Previous research findings implicate that math interest can affect accuracy in numerosity estimation on the condition that students consider estimation as part of mathematics. To date, there has been no study published that examines the connection between math interest and accuracy in numerosity estimation. In this context, we have attempted to contribute some knowledge to this field by specifically examining such a relationship.

We cannot give an unqualified answer to the extent of a relationship between math interest in third-grade students and accuracy in numerosity estimation tasks because our results were inconsistent. For example, we found a significant but weak, negative correlation between the scale *Mix* and math interest. However, the other three scales of accuracy in numerosity estimation were not related to math interest. We had expected either that all scales would correlate with math interest (H_2_) or else that none would if the students did not consider numerosity estimation as mathematical content. So, the pattern of correlations did not support our expectations. In this context, it would be interesting to analyze correlation patterns with a larger sample across more grades, because this result could also be spurious.

In general, we assume that children do not necessarily understand estimation as classical mathematics and, consequently, we cannot conclude a necessary relationship between math interest and accuracy in numerosity estimation. When children were asked about their math interests, they spontaneously appealed to their own experiences in mathematics class.

“Our view of studies on estimation has reviewed how difficult it is for students who received traditional instruction to understand that besides counting precisely and calculating exact answers, there is also something like estimating and developing appropriate procedures and strategies for making appropriate estimate” (Verschaffel et al., 2007, p. 581).

This quote from Verschaffel et al. (2007) supports our stance and illustrates how children associate mathematics with the world of exact numbers until they are shown otherwise, for example in math lessons. Estimation can be seen as a contrast to the world of exact numbers (Schipper, 2009). Therefore, children do not typically associate estimation with mathematics, because estimating requires recalling activities that may never have been experienced in the first place. In this context, the finding that math interest and estimation accuracy are rarely related is not astonishing.

### 4.4. Conclusion

Our results provide new insights into numerosity estimation and its relationship with math achievement and math interest. Numerosity estimation is a neglected area in mathematics education (Andrews et al., 2021), but one that holds high potential for mathematics education in primary school.

The results of this study may have been affected by the specific content of the math achievement test. Specifically, the test’s emphasis on algorithmic calculation may be seen as one limitation of the study. As we mentioned before, estimation requires independent thinking strategies and the skill to solve a problem in a flexible way. These aspects were not explicitly part of the achievement test we used. From a theoretical perspective, we assumed that numerosity estimation influences the development of number knowledge, which is essential for diverse mathematical proficiency (Wessolowski, 2014). Number knowledge includes having a basic idea of numbers that was also not directly addressed in the achievement test. Therefore, it would be interesting to analyze more specific relations between numerosity estimation accuracy and less procedure-based aspects of math achievement. In addition to the analytical methods used, structural equation models are conceivable for future studies examining such relationships.

We did not find a significant relationship between math achievement and estimation accuracy at this point in third grade, but, in our opinion, it would be worthwhile to further investigate this relationship. This study did not aim to answer the question about the influence of fostering numerosity estimation on math achievement over time or if there is a cumulative developmental cycle. So, future research could focus on this influence in longitudinal or intervention studies. Furthermore, we recommend the consideration of two- and three-dimensional numerosity estimation tasks as well other characteristics to focus on commonalities and differences that may influence (components of) math achievement. Since previous research has found that age has an impact on estimation accuracy, it would be equally reasonable to test this relationship at a higher-grade level (e.g., Siegler and Booth, 2005; Luwel and Verschaffel, 2008). The numerosity estimation test we developed could be very useful for longitudinal studies, since it is possible to use the test multiple times. This test is still in the early stages of development and will be further developed and evaluated. One reasonable focus would be the continuing validation of the test and its components.

Our study assessed general math interest. We could not capture situational interest regarding the estimation tasks or interest in estimation. Consequently, modifying or adapting the items on a scale to specify estimation interest would seem to be an important line of further research. It would be worthwhile to investigate, for example, whether estimation interest or situational interest in solving estimation tasks affects the estimation process and thus estimation accuracy and whether such an affect is stable across age groups.

Due to the relevance of estimation for number sense (Sayers et al., 2016), arithmetic achievement (Booth and Siegler, 2006; Wong et al., 2016), and number knowledge (Wessolowski, 2014), it is important to understand exactly how numerosity estimation is connected to math achievement in general, to specific competencies in detail, and to various non-cognitive components. This study provided new insights into this field, and at the same time, it underscores the importance of paying further attention to numerosity estimation in research and fostering estimation abilities in mathematics education.

## Data availability statement

The raw data supporting the conclusions of this article will be made available by the authors, without undue reservation.

## Ethics statement

The studies involving human participants were reviewed and approved by Hessian Ministry of Education. Written informed consent to participate in this study was provided by the participants’ legal guardian/next of kin.

## Author contributions

LB and ER-S contributed to the conception and design of the study. LB organized the database, performed the statistical analysis, and wrote the first draft of the manuscript. ER-S wrote sections of the manuscript and supervised the writing process. All authors contributed to the article and approved the submitted version.

## Conflict of interest

The authors declare that the research was conducted in the absence of any commercial or financial relationships that could be construed as a potential conflict of interest.

## Publisher’s note

All claims expressed in this article are solely those of the authors and do not necessarily represent those of their affiliated organizations, or those of the publisher, the editors and the reviewers. Any product that may be evaluated in this article, or claim that may be made by its manufacturer, is not guaranteed or endorsed by the publisher.
